# Machine learning in attention-deficit/hyperactivity disorder: new approaches toward understanding the neural mechanisms

**DOI:** 10.1038/s41398-023-02536-w

**Published:** 2023-07-01

**Authors:** Meng Cao, Elizabeth Martin, Xiaobo Li

**Affiliations:** 1 Department of Biomedical Engineering, New Jersey Institute of Technology, Newark, NJ USA; 2 Icahn School of Medicine at Mount Sinai, New York, NY USA

**Keywords:** Neuroscience, Biomarkers

## Abstract

Attention-deficit/hyperactivity disorder (ADHD) is a highly prevalent and heterogeneous neurodevelopmental disorder in children and has a high chance of persisting in adulthood. The development of individualized, efficient, and reliable treatment strategies is limited by the lack of understanding of the underlying neural mechanisms. Diverging and inconsistent findings from existing studies suggest that ADHD may be simultaneously associated with multivariate factors across cognitive, genetic, and biological domains. Machine learning algorithms are more capable of detecting complex interactions between multiple variables than conventional statistical methods. Here we present a narrative review of the existing machine learning studies that have contributed to understanding mechanisms underlying ADHD with a focus on behavioral and neurocognitive problems, neurobiological measures including genetic data, structural magnetic resonance imaging (MRI), task-based and resting-state functional MRI (fMRI), electroencephalogram, and functional near-infrared spectroscopy, and prevention and treatment strategies. Implications of machine learning models in ADHD research are discussed. Although increasing evidence suggests that machine learning has potential in studying ADHD, extra precautions are still required when designing machine learning strategies considering the limitations of interpretability and generalization.

## Introduction

Attention-deficit/hyperactivity disorder (ADHD) is one of the most prevalent neurodevelopmental disorders, affecting ~5–8% of children worldwide [1, 2]. For about 60% children with ADHD, the symptoms persist into adulthood [3, 4]. Individuals with ADHD have poorer educational and social outcomes, increased injury incidences during daily activities [5, 6], and an elevated risk of developing more severe mental disorders [7–9]. ADHD is a highly heterogeneous disorder [10]. For example, sex, genetic, and environmental factors have been implicated in the presentation of ADHD [11–13]. There is also diverging evidence regarding the developmental trajectories and comorbidities of individuals with ADHD [14, 15]. Considering the high prevalence and life-long consequences of ADHD, early detection, accurate diagnosis, and efficient treatments are highly desired. However, the field currently lacks a comprehensive understanding of the relevant neural mechanisms and is far from reaching an agreement regarding efficient treatment strategies.

Extensive studies have attempted to characterize ADHD in terms of neuropsychological performance, brain anatomy and functional responses, and genetic risk factors. Cognitive deficits in executive function, reaction time, vigilance, inhibition control, sustained attention, and working memory have been reported in ADHD [16–18]. Neuroimaging studies using T1-weighted magnetic resonance imaging (MRI), functional MRI (fMRI), resting-state fMRI (rs-fMRI), and electroencephalogram (EEG) have reported widespread and inconsistent anatomical and functional alterations in children with ADHD, including frontal lobe, parietal lobe, temporal lobe, thalamus [19–22]. Genome-wide association studies have also revealed several variants associated with ADHD [23–25]. In addition, the treatment of ADHD shows inconsistent results, with evidence suggesting that 30% of ADHD patients respond poorly to the most common ADHD medication [26, 27]. The existing evidence suggests that ADHD may not have a single etiological source but rather a combined effect of multiple subtle anomalies. Such a complex etiology is difficult to detect using parametric statistical methods, and interactions between widespread alterations have not been successfully translated into clinical practice due to the limited capacities of conventional analytical methods.

**Fig. 1:**
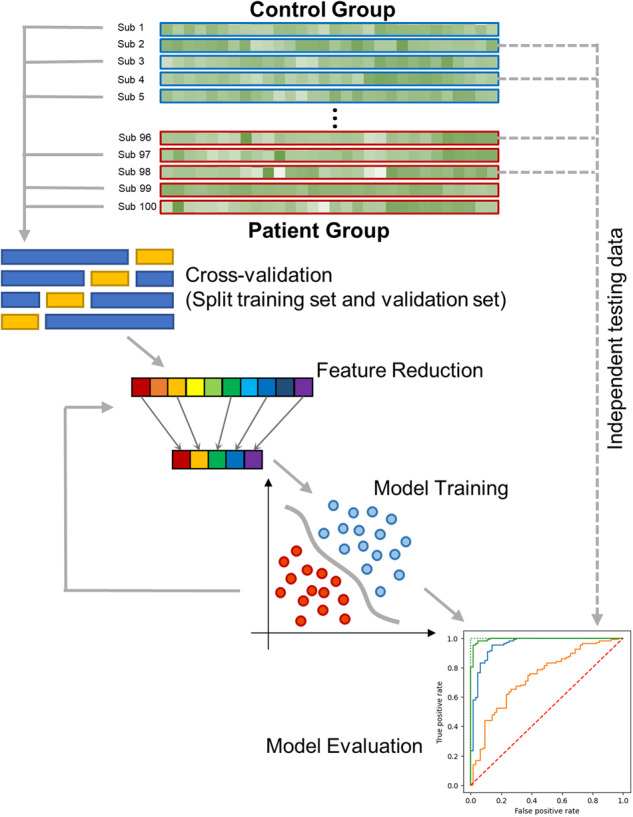
Overview of machine learning steps. Typical steps, including data splitting, feature reduction, and model training, used in machine learning studies of patients with psychiatric conditions.

The increasing accessibility of machine learning models has led to increased interest in applying such models to investigate psychiatric disorders. Generally, machine learning models are mathematical models that learn complex patterns in an existing dataset. These learned patterns can then be used for prediction in a novel dataset (e.g., patient vs control participant, symptom scores), as well as to highlight the most important variables in creating this prediction. Machine learning models have proved effective in capturing the complex interactions between discrete alterations in schizophrenia, Alzheimer’s disease, and autism spectrum disorder (ASD) [28, 29]. Most psychiatric studies have developed models that differentiate patient groups and controls using classification algorithms like SVM, random forest and linear discriminative analysis (LDA). Others predict symptom severity or behavioral performance using regression algorithms, for example, random forest regression, support vector regressor, and elastic net regression. The general steps involve data splitting, feature reduction, and model training, as shown in Fig. 1. The original data is first split into training set (for features selection and training machine learning models), validation set (for validating and tuning parameters of trained models), and testing set (for evaluating the model performance). Before using the training set to train the model, feature reduction is usually performed using feature selection or feature fusion to increase the efficiency of the training process and reduce the chance of overfitting. During the training process, adjustments are made to the model based on the model performance in the validation set. Finally, the effectiveness of the classification models is evaluated in the independent testing set using accuracy, specificity, sensitivity, or area-under-the-curve (AUC), and the performance of the regression models is evaluated by the mean square error or the correlation [30]. However, many studies to date report model performance based on the results of cross-validation processes without having independent set testing. Such studies can yield less reliable or less generalizable results, and therefore, extra precautions are needed when interpreting the findings of these studies. Using this general process, most machine learning studies have been able to differentiate patients with psychiatric disorders and controls with AUC from 60 to 90% [29]. For diagnostic purposes, models with an AUC of less than 60% were considered to have bad performance, while models with an AUC of more than 80% were considered as having very good performance [31].

Increasing evidence suggests that machine learning techniques are beneficial in improving ADHD diagnosis, understanding neurobiological substrates, and evaluating treatment strategies (for reviews [32–35]). For example, current diagnoses of ADHD require extensive interviews of parents and teachers (for childhood ADHD) and of patients (for adult ADHD) on observations of current and past ADHD symptoms and subsequent impairment of daily functioning. Machine learning studies can learn from sufficient samples to sort and select the most relevant interview questions for accurate diagnoses. Therefore, machine learning has the potential to facilitate the development of more efficient diagnostic procedures for ADHD. Additionally, the ability to predict treatment outcomes using machine learning may contribute to the emergence of precision medicine (for reviews on these topics, see [32–35]). The abundance of machine learning investigations in neuroimaging studies has partially been enabled by the public release of the ADHD-200 dataset [36], which has allowed exploration in automating ADHD diagnosis [35]. Recent public datasets like Adolescent Brain Cognitive Development (ABCD) dataset also boosted the machine learning research in ADHD [37].

The majority of existing machine learning studies in ADHD focus on developing classification algorithms between ADHD patients and controls or patients with other comorbid disorders. Undoubtedly, machine learning algorithms were best suited for predictive purposes. However, the sample size is often a study limitation. Large and high-quality datasets are difficult to obtain due to the substantial efforts required in the collection and maintenance processes. Studies in small-size samples tend to get over positive results and less generalizable models without including appropriate validation processes (e.g., lack of independent validation set or leakage between the validation set and training set) [38, 39]. For example, leakage between the training set and validation/testing set, or feature selection/reduction before data splitting, can lead to substantial bias in the machine learning models [40]. Despite these issues, there are merits of studies with smaller sample sizes. Relative to large sample studies, small sample studies may be able to recruit more homogeneous groups. In these homogeneous samples, after proper validation and evaluation process, the inference of important features, rather than building accurate classification models, is more beneficial to the research in ADHD. Due to the ability of machine learning models to extensively learn the complex patterns in a dataset, they can also be used to compare different modalities or identify important features. Be advised that current methods for calculating feature importance still have limitations. For example, the most advanced or complex models don’t inherently rank feature importance; and generalized feature importance scores may not describe the true relationship that is utilized in the models [41, 42]. Additionally, many studies (despite reporting feature importance scores) aim at demonstrating machine learning models with high classification accuracy rather than understanding the most representative biological information.

Existing reviews have summarized the effectiveness of different machine learning models in differentiating subjects with ADHD from control subjects or subjects with other disorders [32–35]. However, in addition to building classification models to aid diagnosis, machine learning is advantageous in studying mechanisms underlying ADHD due to its ability to describe the vast heterogeneity in the etiology of ADHD. The purpose of the present narrative review is to summarize the current literature regarding the applications and benefits of using machine learning algorithms to understand the underlying neural mechanisms of ADHD, as well as ongoing issues and future research directions. Although the aforementioned limitations of the interpretation of feature importance exist, exploring the possible applications may lead to the development of feature-focused and explainable machine learning models in ADHD. Studies were included if they met the criteria of (1) using machine learning algorithms, (2) having a total sample size of at least 40, (3) applying a cross-validation step, (4) model evaluation is independent of model training, (5) reporting comparisons between features (e.g., feature importance, or performances of different measuring modalities). A full list of search terms used in this review can be found in the Supplementary document. An overview of the studies that applied machine learning algorithms in investigating ADHD is presented in Supplementary Fig. 1. The detailed methodology and key findings of the included studies can be found in Supplementary Tables 1–4.

## Machine learning in characterizing ADHD

The use of machine learning in aiding the diagnosis of ADHD has been covered extensively in existing reviews [32, 34, 35] and will, therefore, not be discussed in detail in this review. Briefly, some evidence suggests that machine learning algorithms have the potential to benefit the diagnosis of ADHD by either simplifying the diagnostic process in complex cases (e.g., achieving similar accuracy with less items, increasing accuracy in patients with comorbidities) [43–49] or increasing accuracy with additional neurobehavioral measures or activity records [50–55]. The contribution of the classification models can be limited by factors such as the sample sizes used, which often contribute toward inflated accuracies. Instead, by inspecting the features identified as most important in classification models, machine learning algorithms were able to identify the core characteristic of ADHD.

A recent nationwide study in Sweden applied multiple machine learning models, including random forest, elastic net, deep neural network, and gradient boosting, in identifying the significant predictors for ADHD based on family and medical histories from 238,696 individuals [56]. The best model achieved a sensitivity of 71.7% and a specificity of 65.0%, and the results showed that the top risk factors for ADHD in children are having parents with criminal convictions, male sex, having a relative with ADHD, academic difficulties, and learning disabilities. Another study investigated Conner’s rating scale from both parents and teachers in differentiating children with ADHD and controls using a deep neural network [57]. The models demonstrated an accuracy of 89%. More interestingly, the study reported that teachers’ ratings for the oppositional questions were more discriminative for ADHD than parents’ ratings. In addition, questions directly describing the symptoms were more discriminative than the question worded metaphorically. Among adults with ADHD, one study with 1249 subjects reported that difficulty organizing, does not follow through, making careless mistakes, and difficulty engaging in leisure activities were key characteristics of adult ADHD [58]. This evidence from existing machine learning studies may expand the understanding of the characteristics of ADHD and provide guidance for developing more reliable and efficient diagnostic criteria.

Beyond allowing the classification of subjects into traditional diagnostic groups, research into machine learning-aided diagnosis of ADHD has contributed to the understanding of the clinical presentation and heterogeneity of ADHD by allowing the identification of novel subgroupings of participants, which can increase diagnostic accuracies [59, 60]. For example, Fair et al. evaluated the performance data during seven neuropsychological tasks, including inhibition, working memory, arousal, response variability, temporal information processing, memory span, and processing speed, in a cohort of 285 children with ADHD and 213 controls [61]. By implementing community detection methods, four subgroups in both the ADHD group and the control group were identified. Classification using SVM following this subgrouping led to a diagnostic accuracy as high as 84.1%, compared to a markedly lower classification accuracy of 65% without subgrouping. Similarly, Kleinman et al. regrouped healthy children and children with ADHD, bipolar disorder, or both into two groups based on continuous performance task (CPT) performance [62]. LDA was then used to build separate classification models on both the Diagnostic and Statistical Manual of Mental Disorder (DSM) IV-based groups and CPT-defined groups. CPT-defined groups had a markedly higher discriminative accuracy (95.2%) than the DSM IV-defined groups (23.8%). A more recent study performed clustering analysis in a combined group of children with ADHD, children with ASD, and controls based on the behavioral measure from 12 domains [63]. Three executive function-defined groups were detected, including weakness in flexibility and emotion regulation, weakness in inhibitory control, and weakness in working memory, organization, and planning. SVM was used to validate the detected subtypes in an independent dataset and yield a classification accuracy of 88.9%. Within a subset of the subjects, the detected subgroups explained more between subject variance than the DSM-defined clinical groups. Such studies suggest that although existing clinical classifications may be sufficient to identify ADHD, they cannot comprehensively capture the heterogeneities.

In general, the accuracy of the classification models varies from 66 to 96% in the existing machine learning studies that investigated behavioral and cognitive performances in ADHD. The inconsistency was partially contributed by the differences in total sample size, percentage of the clinical group in the total sample, test or measurement selection, model selection, or validation methods. Therefore, extra precaution was required in designing reliable classification models. Furthermore, machine learning techniques that can explore the heterogeneities in ADHD (e.g., clustering analysis, regression analysis) may not only improve diagnosis but may contribute to improvements in future research investigating the underlying mechanisms by providing more appropriately defined samples.

## Machine learning in investigating biological mechanisms of ADHD

### Neuroimaging studies

#### Structural MRI and diffusion tensor imaging

The neuroanatomy of ADHD has been investigated for decades. However, results are inconsistent [19, 64, 65]. A recent mega-analysis reported subtle alterations in surface area in various cortical regions in ADHD [20]. Studies using diffusion tensor imaging (DTI), a neuroimaging technique that measures microstructural changes, also reported white matter alterations in widespread regions [66]. This existing evidence suggests that ADHD might not be related to highly localized anatomical alterations but more diffuse changes [67–69]. Existing research may be limited by the use of conventional statistical methods, which lack sensitivity to subtle changes over multiple regions and the interactions between them.

Machine learning, on the other hand, can model a number of features simultaneously, making machine learning approaches particularly well-suited to understanding the widespread structural alterations underpinning ADHD. For example, Peng et al. reported results from an extreme learning machine-based classification model which differentiated children with ADHD and controls with an accuracy of 90.18% using sMRI data from ADHD-200 [70]. The model identified surface area, folding index, and volume in the parietal lobe, temporal lobe, and insula as the most important predictors of ADHD. Another study using SVM for classification showed that the white matter volume in the brain stem was the most important feature in differentiating boys with ADHD and controls [71]. Using LASSO regression, a recent DTI study reported that the tract strength between the substantia nigra/ventral tegmental area and the striatum was able to predict impulsivity with a Spearman’s correlation of 0.17 in a group of 74 ADHD patients and controls [72]. In a large cohort (4183 subjects from 35 study sites), deep learning neural network revealed that sMRI was a good predictor of ADHD in children but not in adults, supporting the idea that structural alterations associated with ADHD lessen with age [73]. Studies using sMRI can also identify structural properties that distinguish ADHD from other common disorders. For example, Lim et al. investigated the discriminative power of structural properties in ADHD, ASD, and control participants [74]. With voxel-level gray matter volume as features, a Gaussian process classification algorithm differentiated ADHD specifically (compared to ASD) from controls with 79.3% accuracy and highlighted several regions in which structural properties contributed highly to this classification. Those regions may be involved specifically in the pathophysiology of ADHD, as opposed to ASD. Despite these promising results, Oztekin et al. found that parent and teacher ratings of executive function in an SVM model resulted in an accuracy of 92.6%, while using sMRI data alone resulted in an accuracy of 61.2%, and adding anatomical features to a model containing neurocognitive measures had minimal benefit [75]. Therefore, in some cases, the additional benefit of sMRI measures for classification may be limited, although they can still contribute toward identifying underlying structural differences.

Machine learning can also be used to explore novel sMRI features, which may provide optimal discriminative power for ongoing research into ADHD. For example, Chang et al. generated novel morphological features based on the local binary patterns (an image texture categorization method) to differentiate data from 210 ADHD and 226 controls from the ADHD-200 dataset [76]. An SVM model applied to the generated features achieved an accuracy of 69.95% in detecting ADHD. Similarly, using volumetric features named Dissociated Dipoles, Igual et al. built an SVM-based classification model with an accuracy of 72.48%, a specificity of 85.93%, and a sensitivity of 60.07% [77]. Another team used a hybrid machine learning approach on novel interregional morphological connectivity features and reported a classification accuracy of 74.65% [78]. Although currently, these studies do not contribute to our understanding of anatomical alterations in ADHD per se, they contribute to the field by highlighting features that may be beneficial for improved diagnosis or sample classification.

#### Task-based fMRI

**Fig. 2:**
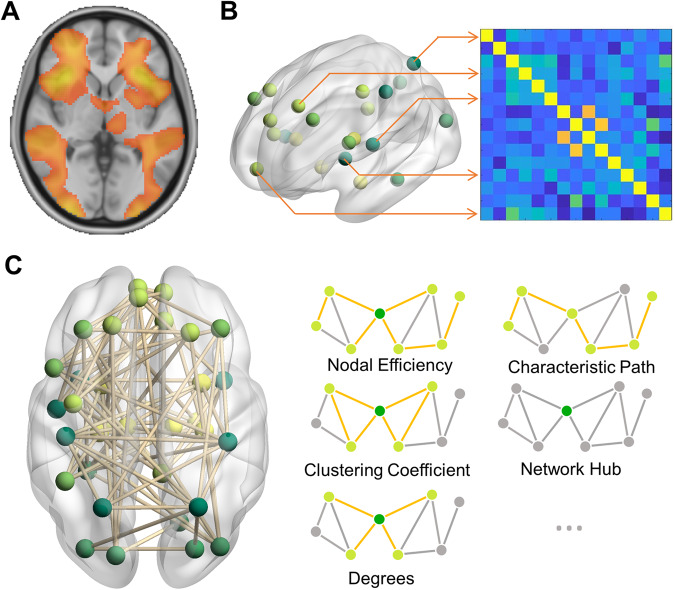
Functional neuroimaging features. **A** Voxel-level features, for example, voxel-level activation in task-based fMRI or regional homogeneity in resting-state fMRI. **B** Functional connectivity. **C** Network topological properties in graph theoretical analysis. fMRI functional magnetic resonance imaging.

Task-based fMRI is a commonly used method to examine brain activation or functional connectivity during of engagement of a specific cognitive domain. Features like voxel-level activation, functional connectivity between regions-of-interest (ROIs), or network topological properties (as shown in Fig. 2) can be used to build machine learning models. Several studies have applied machine learning techniques to fMRI data collected from participants with ADHD. For example, by applying various machine learning algorithms to the functional activations during time discrimination tasks [79], Flanker tasks [80], and stop-signal task [81], studies have highlighted that the task-related activations in frontal regions were important for the classification of ADHD, suggesting functional importance of frontal regions in ADHD.

Importantly, machine learning algorithms may be able to detect functional patterns (e.g., the collective contribution of multiple brain regions in differentiating ADHD and controls), which may otherwise be undetected when using traditional methods. For example, Wolfers et al. applied a Gaussian process classifier in differentiating subjects with ADHD, their unaffected siblings, and controls based on the fMRI data during stop-signal task [81]. The model was able to differentiate ADHD patients from their siblings with an AUC of 0.65 and from control participants with an AUC of 0.64. The results showed that the fronto-lateral and inferior parietal regions were highly discriminative features for ADHD. Hart et al. utilized a Gaussian process classifier to differentiate boys with ADHD from controls based on fMRI data recorded during a stop-signal task (used to measure response inhibition) [82]. Using voxel-level functional activation as feature, the classification accuracy reached 77%. Interestingly, voxels that showed no significant group differences using traditional univariate analysis demonstrated high discriminative power when using machine learning, suggesting that machine learning methods can tease out important discriminatory activations above and beyond traditional analysis methods.

#### Resting-state fMRI

The brain demonstrates intrinsic spontaneous activity that can be measured during rest. rs-fMRI measures such activity, and the collected data can be used to generate machine learning features, such as regional homogeneity (ReHo), fractional amplitude of low-frequency fluctuation (fALFF), and network connectivity. As rs-fMRI data does not require the performance of a task, it is easy to implement in children with ADHD. Classification techniques have highlighted regions in which resting brain activity is of potential importance in ADHD. For example, studies using SVM have revealed that functional connectivity in default mode network, frontoparietal regions, cerebellum, precuneus/posterior cingulate cortex regions, and dorsal anterior cingulate cortex were important in differentiating ADHD [83, 84].

As previously mentioned, the ADHD-200 dataset has allowed numerous investigations into rs-fMRI correlates of ADHD using machine learning algorithms. Various rs-fMRI features have been explored, including ReHo, fALFF, power spectra, functional connectivity, and voxel- and ROI-level functional networks [85–87]. Eloyan et al. constructed a classification algorithm based on majority voting from four algorithms, including random forest on motor cortex connectivity, SVM on major clusters, gradient boosting method on decomposed functional connectivity, and gradient boosting on functional connectivity and motion parameters [88]. The final model achieved a specificity of 94% and a sensitivity of 21%, and connectivity within the motor network was most important in classifying ADHD participants. Several studies have utilized SVM to construct classification models and report that the frontal lobe, parietal lobe, and cerebellum are most discriminative between ADHD and controls and between ADHD inattentive subtype and ADHD combined subtype [89, 90]. Similarly, a graph convolutional neural network study identified the frontal, temporal, and occipital regions and the cerebellum as the most discriminative regions for ADHD and controls [91].

Despite the success of rs-fMRI-based machine learning models, it is possible that phenotypic information such as gender, age, and cognitive measures provide more discriminative power than rs-fMRI data [92, 93]. However, the addition of rs-fMRI features may be beneficial nonetheless. For example, Bohland et al. found that the addition of such features increased generalization to novel data [93]. Additionally, studies have suggested that rs-fMRI data are more predictive for inattentive symptoms rather than hyperactive/impulsive symptoms [94] and that classification accuracy increases when using an SVM trained separately for male and female subjects [95], reflecting that certain applications of such models can yield more accurate results. Such considerations may be useful in future rs-fMRI research.

#### EEG

**Fig. 3:**
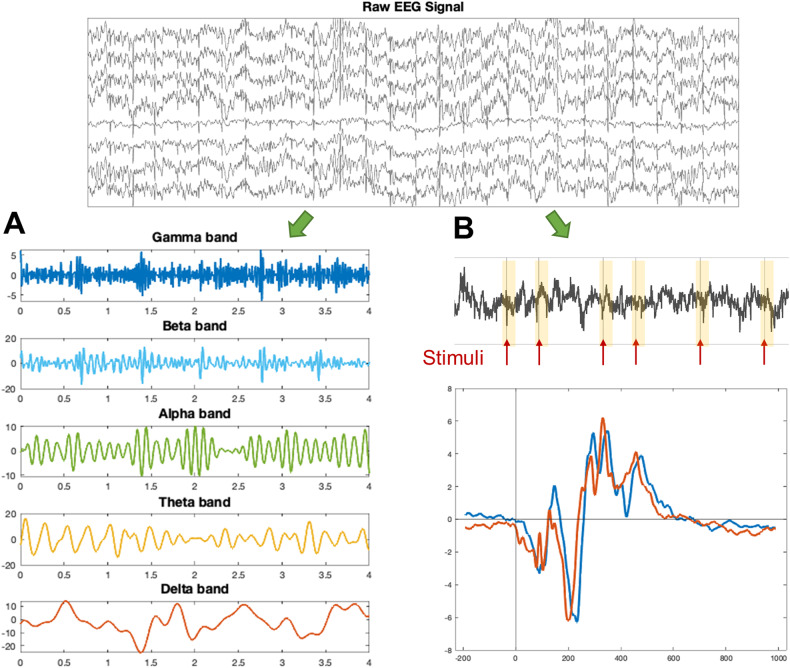
Electroencephalogram features. **A** Frequency analysis, including power at different frequency bands. **B** Event-related potential analysis, including the time or amplitude of peaks after the target stimuli.

Due to its high accessibility, low cost, and non-invasive nature, EEG has gained popularity in studying ADHD. Common features generated from EEG data are power in frequency bands at different locations and event-related potentials (ERPs), which are electrical responses that are time-locked to the occurrence of sensory or cognitive processes, as shown in Fig. 3. Several studies using machine learning have shown that features extracted from EEG data can be used to differentiate ADHD patients from controls and from other comorbid conditions with varied accuracy ranging between 69 and 91% [96–99]. Classification of specific diagnostic subtypes of ADHD based on EEG features is also possible, although with a lower classification accuracy of around 72% [100, 101].

Several studies have investigated the predictive power of specific features of EEG data. For example, using deep neural network, one study identified that ERPs within the time range from 100 to 200 ms post-stimulus are important in differentiating children with ADHD and controls during an interval-time task [99]. The model was able to differentiate the ADHD group and controls with an accuracy of 69%. In addition, several factors appear to contribute to the accuracy of EEG-based models. Several studies have assessed the optimum experimental paradigm for classification. For example, Chang et al. reported that the signal during the transition period between the task and resting condition was more discriminative for ADHD than the signal during the task condition or resting condition [102]. Tenev et al. reported that a model combining multiple task conditions showed a significant increase in classification accuracy when compared with a single condition (82.3% vs 70%) [103]. Studies using the go/no-go task report inconsistent results regarding the most discriminative task conditions. For example, Mueller et al. reported that an ERP-based network for No-go had significantly higher predictive power than that during the Go condition for a visual sustained attention task [104]. However, Biederman et al. reported that an SVM-based model using the signal from the Go condition achieved higher AUC than the signal during the No-go condition (0.92 vs 0.84) [105]. Age may also be an important influence on classification. For example, splitting subjects into different age groups increased classification accuracy when applying SVM on EEG data [106].

Machine learning is also valuable in investigating novel EEG features. For example, Kim et al. used machine learning to validate mismatch negativity (a novel measure that contrasts activity during regular auditory stimuli and occasional novel stimuli) in differentiating adults with ADHD from controls [107]. The SVM-based model showed a classification accuracy of 81% and identified the frontal lobe, temporal lobe, and limbic lobe as the most important regions in the classification. Studies have also constructed machine learning models using other novel features, including various entropy-based features and fractal dimension-based features from chaotic theory [108–110]. As research continues to employ machine learning methods, it is likely that novel features to best classify individuals with ADHD will continue to be determined.

#### Functional near-infrared spectroscopy

Functional near-infrared spectroscopy (fNIRS) is a non-invasive and portable method to measure the hemodynamic response in the cortex. Relative to fMRI, fNIRS is less susceptible to the movements and is therefore well-suited to study ADHD, and machine learning has the potential to utilize the fNIRS’s high temporal resolution while overcoming its low spatial resolution. One study applied SVM on fNIRS data from children with ADHD and controls during a working memory task [111]. The final model achieved an accuracy of 96% and highlighted the dorsal lateral prefrontal cortex, temporal cortex, medial prefrontal cortex, and posterior prefrontal cortex as the most discriminative in classifying ADHD and controls. Yasumura et al. applied an SVM-based model on fNIRS data from children with ADHD and controls collected during a reverse Stroop task [112]. The model achieved 86.25% accuracy with a sensitivity of 88.71% and a specificity of 83.78%. Splitting the sample into three age groups (<10 years, 10–12 years, >12 years) increased classification accuracy significantly.

#### Multimodal imaging

Given its ability to model several features simultaneously, machine learning is well-suited to multimodal investigations of neural markers of ADHD. For example, Zhou et al. combined rs-fMRI with sMRI and DTI data from the ABCD dataset and reported that the functional connectivity in frontal and temporal regions, cerebellum, thalamus, and anatomical regions in the basal ganglia were the most discriminative features for ADHD in children [113]. Luo et al. utilized multimodal imaging data, including fMRI data during a cued attention task, sMRI, and diffusion tensor imaging [114]. The algorithms combined a range of machine learning models and achieved an accuracy of 89% in differentiating adults with ADHD and controls and an accuracy of 90% in differentiating ADHD persisters and remitters. The results showed that functional connectivity in the frontal and parietal lobe and amygdala volume was important to differentiate ADHD with controls, while functional connectivity in the frontal lobe, parietal lobe, and putamen was important to differentiate ADHD persisters and remitters. Owens et al. combined task-based fMRI data and structural MRI data from the ABCD dataset to investigate the relationship between ADHD symptoms and imaging measures [115]. Using the elastic net algorithm, results showed that, compared to other modalities, functional activation during a working memory task can predict ADHD symptoms with the best performance, which explained 2% of the variance with a small effect size. Combining multimodal data offers the opportunity to identify a range of biomarkers, which is particularly advantageous in ADHD, given its complex etiology.

### Genetic studies

#### Genetic studies

Genetic and twin studies suggest that ADHD is highly heritable [116–119]. This heritability may be due to polygenic risk [120, 121]. Recent genome-wide association studies provide promising results in understanding the genetic associations with ADHD [25]. Machine learning handles multiple independent variables simultaneously, allowing the interactions between various risk factors to be assessed. In addition, it highlights risk factors that are statistically insignificant but may contribute to ADHD. These properties make machine learning a particularly valuable tool in studying genetic markers of ADHD.

van der Meer et al. used a random forest regression model to investigate the predictive power of 29 stress-related genes on ADHD severity in children with ADHD, subthreshold ADHD, and controls [122]. The model explained 12.5% of the variance in ADHD severity and indicated that, besides chronic stressors, the region that regulates the expression of telomerase reverse transcriptase was important in predicting ADHD severity. Other studies have used random forest and convolutional neural networks to study genetic predictors of ADHD and have revealed that the gene regions GRM1, GRM8, and EPHA5 are important predictors of ADHD [123, 124]. Using multiple machine learning algorithms, a recent study reported that age and sex were significant predictors in genetic information-based classification models [125]. In addition, gene regions SNAP25, ADGRL3, and DRD4 significantly contributed to the prediction of inattentive, hyperactive, or impulsive symptoms. SVM models have also shown that microRNA has high discriminative power for ADHD and can predict medication responses in ADHD patients [126].

#### Multi-omics studies

Machine learning algorithms allow the combination of genetic data with data such as cognitive and neuroimaging data. For example, using conditional random forests, Sudre et al. were able to predict ADHD severity with an AUC of 0.79 [127]. While cognitive measures were most important in the overall classification, genomics was important in detecting children with worsening ADHD, highlighting the utility of multimodal machine learning approaches. Yoo et al. combined anatomical features from both sMRI and DTI, functional connectivity during rs-fMRI, and genetic data related to norepinephrine, dopamine, and glutamate to build a random forest-based classification model and regression model for ADHD [128]. The classification model using cortical thickness and volumes achieved the best performance with an accuracy of 85.1% and an AUC of 0.877 in differentiating ADHD participants and controls. Additionally, the regression model was able to explain 18% of the variance of the ADHD rating scale. Both models did not gain improvements when including genetic data. Future machine learning studies may be needed to further investigate the relations between genetic data, neurocognitive performance, behavioral problems, and neurobiological alterations in ADHD patients.

## Machine learning in predicting treatment and prognostic outcomes of ADHD

Heterogeneity in ADHD imposes difficulties in developing effective and reliable treatment strategies. Methylphenidate (MPH) is one of the main pharmacological treatments for ADHD; however, 30% of patients are poor responders [26, 27]. Machine learning techniques are beneficial to predicting treatment outcomes as they have the ability to provide predictions from relatively little prior knowledge. Several studies have predicted response to MPH using SVM, with features including neuropsychological test performance and information on clinical information [129] and sMRI data [130]. Faraone et al. implemented lasso regression to predict the responses of adolescents to a novel non-stimulant medication (SPN-812) [131]. Responder status (with good responder defined as a >50% improvement in symptoms score) after 6 weeks was predicted with the response data (symptom score change from baseline) collected up to weeks 1, 2, and 3. The lasso regression model predicted the long-term result based on the outcome at 2 weeks with 75% accuracy.

Machine learning can also be utilized to predict adverse drug outcomes, which are common in ADHD treatment. For example, Yoo et al. predicted sleep side effects of using MPH treatment based on multiple variables and achieved an accuracy of 95.5% [132]. Based on findings using long short-term memory model, Fouladvand et al. reported that the initiation of ADHD medication during adolescence is a significant predictor for developing substance use disorder in a large cohort with 11,624 children with ADHD [133]. Zhang-James et al. also reported ADHD medication as one of the important predictors of substance use disorder, along with ADHD diagnosis before 12 years old and crime behaviors [134]. Given that finding the most suitable ADHD treatment is largely still dependent on trial-and-error of medications and the risk for adverse outcomes of drug treatment, the ability to predict treatment outcomes using machine learning models has the potential to reduce financial and medical burdens.

## Discussion

A growing number of studies are utilizing machine learning techniques to report interpretable results regarding neural mechanisms associated with ADHD, in addition to building accurate classification models. Such studies have already contributed to the literature regarding functional, structural, and physiological correlates of ADHD.

### Performance of machine learning models

A particular benefit of classification models is the ability to label individuals. In addition to the detection of important features, machine learning can assist the development of individualized treatment plans for ADHD (e.g., [130, 135]). The idea of precision medicine has been introduced and practiced in many other diseases [136, 137]. Machine learning studies can accelerate this process in ADHD. This application will no doubt further benefit from the increasing accessibility of large datasets (e.g., ABCD dataset, Human brain mapping dataset, and UK Biobank dataset), which can be used to train more reliable classification models. Groups with small cohorts can also benefit from collaborations with other groups like The Enhancing NeuroImaging Genetics through Meta-Analysis (ENIGMA) Consortium [138]. Alternatively, He et al. also proposed meta-matching methods to utilize information generated from large public datasets when working on independent datasets [139]. The construction of a reliable model using such data could dramatically reduce the workloads of clinicians, thereby increasing the capacities of the existing medical system and minimizing the burden on affected families and societies.

The existing classification models for ADHD reported largely inconsistent accuracy, with the majority varying from 60 to 90%. Several factors related to machine learning design may contribute to these discrepancies. First, the choice of machine learning algorithms may affect the performance of the classification model based on different datasets. Algorithms with very few or no trainable parameters were preferable for studies with small sample sizes, whereas studies with large datasets were able to explore the effectiveness of deep learning algorithms [56, 73, 133]. The second factor is the size balance between different groups. Most studies were able to recruit patient groups and control groups of relatively similar sizes. However, for clinical studies or population-based studies, the balance is hard to achieve [56, 58, 115]. This may potentially give overly positive results. For example, the same model may have a much higher AUC in a population sample than in a group-matched clinical sample (AUC: 0.86 vs 0.72) [140]. Lastly, the choice of validation-test strategy may contribute to inconsistencies in accuracy. A large independent testing set is the best choice for testing generalizability but is only affordable for studies with large datasets [115, 133]. Nested cross-validation may be an alternative, in which the inner cross-validation layer is responsible for training algorithm’s parameter, and the outer cross-validation layer is solely responsible for performance evaluation [71, 113, 114]. However, more than half of the existing machine learning studies in ADHD have only reported results using only one cross-validation, which can cause overfitting of the features and reduce the generalizability of the results.

### Identification of important features

A major benefit of machine learning techniques is that they always involve multivariate data, and some machine learning models like SVM and random forest can rank the contribution of features under the interaction of each other [141]. Therefore, the important clinical or biological features in identifying ADHD can be evaluated based on their contribution to the model. Accuracy (or AUC) can also be used to compare the effectiveness of different feature sets. The training process of a machine learning model extensively learns the information associated with the classification labels within the dataset. When training the same model with features from different modalities, accuracies can partially reflect the sensitivity of particular modalities in ADHD. This evidence can be used to guide experimental design in future hypothesis-driven research.

However, several factors limit the interpretability of the important features reported in existing machine learning studies. First, not all of the machine learning models have intrinsic operations to rank the input features for their importance during the learning procedures. Although generalized feature importance methods exist, such as permutation importance, these methods do not necessarily represent the covariate information used in the original models [41, 142]. On the other hand, for machine learning models that include feature ranking mechanisms, the reported results can be restricted by the ranking methods of the models. For example, feature ranking in a linear SVM only recognizes the high contribution features that show linear relationships with ADHD. Additionally, the majority of the existing studies have only focused on reporting learning procedures that achieved high classification accuracies without giving enough consideration to the “biological meaningfulness” of the study features. Lastly, the field still lacks gold standards in evaluating the quality of a machine learning study. For example, the studies in this review have reported model performance based on different evaluation methods, like cross-validation, nested cross-validation, or independent testing set, using various metrics, including accuracy, AUC, specificity, and sensitivity, meaning the performance may not be comparable. Authors may choose the favorable metrics that do not represent the true performance, and interpretation of the feature importance of such overfitted or biased studies requires extra precaution.

### Current challenges

Despite great promise, challenges are also present before machine learning can provide significant clinical benefits for ADHD due to its heterogeneity. First, machine learning algorithms currently lack interpretability. High-accuracy models are usually constructed with a collection of variables [91, 114, 122], with each variable contributing partial information in distinguishing subjects. The relationship between variables is hard to characterize. Currently, one can rely on the feature’s importance score to provide future direction in investigating particular measures. Models that can translate complex interactions between objective measures are truly beneficial in understanding the neural mechanisms associated with ADHD. A second challenge is the limited generalizability of classification models trained on small samples. Although most studies reported here implemented cross-validation methods to combat overfitting and generalization problems, the nature of the imbalances in the number of features vs the number of subjects in clinical studies and the high heterogeneity of study samples still impose limitations on generalizability [143]. Notably, classification accuracy can drop significantly when applying a trained model to new subjects [45, 46], highlighting the critical need to overcome the generalization problems when implementing machine learning.

### Future directions

**Fig. 4:**
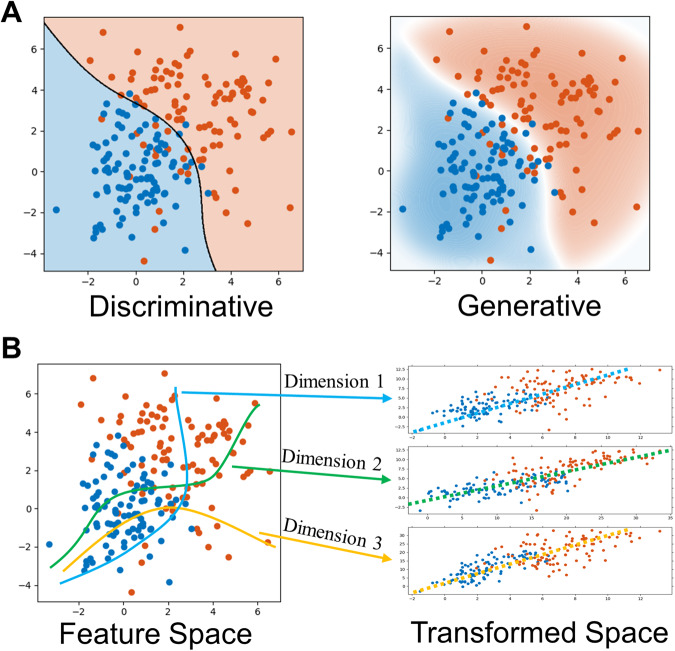
Generative approaches and dimensional approaches for machine learning studies. **A** Discriminative model vs generative model for classification between two groups. Discriminative models aim to define the clear boundary between different groups, while generative models aim to characterize each group and classify based on probability or likelihood. **B** Dimensional approaches. Regression models are used to study the relationship between the target clinical measures and data in transformed dimensions.

Machine learning techniques are still currently undergoing extensive development. Several directions have the potential to resolve the existing problems. One direction is taking a generative approach. Most existing machine learning studies have utilized discriminative models focused on finding the boundaries between known groups within a sample. On the contrary, generative models focus on characterizing groups and predicting group allocation based on probability, as shown in Fig. 4A. In addition, generative models can also characterize samples by identifying subgroups that cluster together. Considering that ADHD diagnosis is usually based on subjective measures and that comorbidities are frequently observed, a hard boundary in the classification process may not be an appropriate threshold for ADHD. Within the literature reviewed here, discriminative models were more effective in constructing accurate classification models (e.g., [75, 111, 114].). This is likely due to the more homogeneous sample using carefully selected inclusion and exclusion criteria [39]. Compared to discriminative models, generative models are less vulnerable to the bias induced in the dataset and therefore can generalize well. In addition, due to the heterogeneous nature of mental disorders, there might be multiple etiological sources or various clinical profiles. Generative unsupervised learning models can detect the homogeneous subtypes otherwise hidden to traditional statistical methods [144]. This property opens the opportunities to capture the heterogeneities embedded in ADHD. More importantly, more homogeneous subgroups expand the interpretability of important features.

Another direction is taking a dimensional approach. The categorical definition of ADHD may not be sufficient to describe the ADHD symptoms in a comprehensive way. Fair et al. reported distinct ADHD subgroups based on cognitive performance, suggesting that neurobiological properties of ADHD might need to be characterized using multiple cognitive measures in addition to the DSM-based symptom measures [61]. Using regression-based machine learning algorithms to associate biological features to multiple clinical dimensions simultaneously can link heterogeneities in both clinical presentations and biological properties of ADHD, therefore increasing the interpretability, for example, in Fig. 4B. This dimensional direction is in line with the National Institute of Mental Health Research Diagnostic Criteria (RDoC) project, which introduced a framework to eliminate diagnosis-imposed boundaries [145]. Notably, several studies have defined the clinical groups based on cognitive or behavioral profiles, and all yield more distinctive groupings than traditional DSM clinical groupings [62, 63, 146–148]. As deficits can be explained along several dimensions (for example, attention, cognitive control, or perception constructs in the RDoC matrix), it may therefore be easier to link to the related biological substrates. In addition, this brings opportunities to explore the phenotypes or endophenotypes of ADHD and explain the heterogeneities in current findings.

In summary, early attempts to investigate ADHD using machine learning show promising results. In addition to seeking high classification accuracy, studies using machine learning to study ADHD can identify the importance of features and discriminative power of modalities, which provide clinical and research targets. Future studies focusing on increasing the interpretability and generalizability of models are highly desired.

## Supplementary information

Supplemental material
