# Supplementary material for: Machine learning in attention-deficit/hyperactivity disorder: new approaches toward understanding the neural mechanisms
Source: Transl Psychiatry. 2023 Jul 1;13:236. doi: 10.1038/s41398-023-02536-w (PMC10313824; doi:10.1038/s41398-023-02536-w)
Supplement: Supplementary file 1 — Supplemental material [file 41398_2023_2536_MOESM1_ESM.docx]

**Machine Learning in Attention Deficit/Hyperactivity Disorder: New Approaches Towards Understanding the Neural Mechanisms**

**Meng Cao, Elizabeth Martin, Xiaobo Li***

**Supplementary Documents**

**Supplementary Document 1. Search terms**

The literature search was conducted in PubMed repository using the following terms in abstract and title:

((ADHD) OR (Attention Deficit Hyperactivity Disorder)) AND ((Classify) OR (Classification) OR (Predict) OR (Prediction) OR (Recognition) OR (Regression) OR (Machine Learning) OR (Deep Learning) OR (Supervised learning) OR (unsupervised learning))

**Supplementary Figure 1. The factions of different types of study after initial screening.**
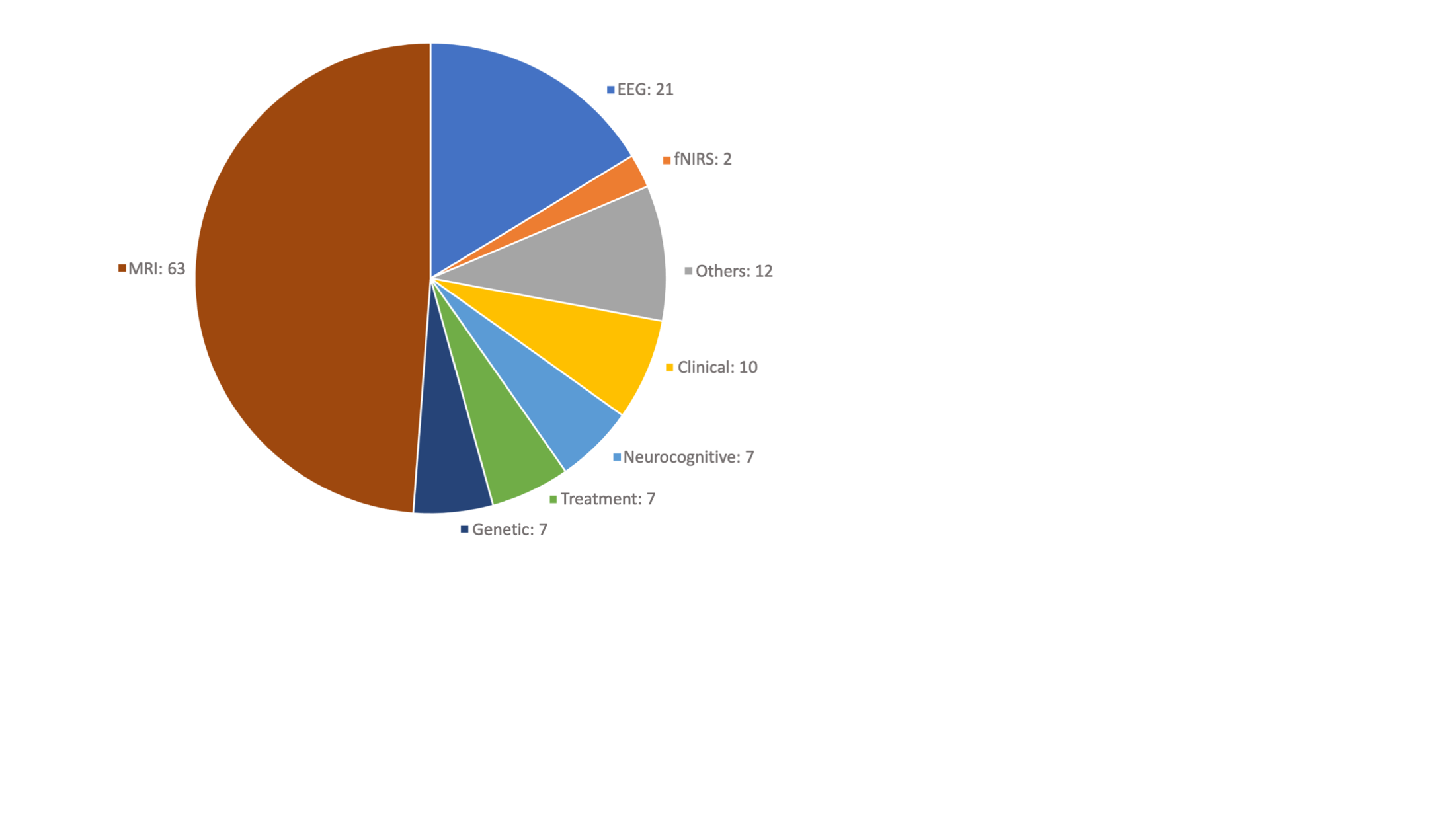


**Supplementary Table 1**. Behavioral and cognitive studies using machine learning.

| **Study** | **Age (Year)** | **Sample Size** | **Features** | **Machine Learning Model** | **Cross-validation Method** | **Independent Testing Dataset** | **Model Performance** | **Key Findings** |
| --- | --- | --- | --- | --- | --- | --- | --- | --- |
| Fair et al., 2012 | 6-17 | 498,  -ADHD: 285,  -Control: 213 | Cognitive | Community Detection, SVM | LOO-CV | 50% of the original sample | 4 clusters identified,  Acc: 61.9-84.1% | Separating subjects into distinct neuropsychological subgroups can increase the classification accuracy |
| Kleinman et al., 2015 | 12-17 | 84,  -ADHD: 23,  -Control: 18,  -ADHD&BD: 33,  -BD: 10 | CPT | k-mean cluster, LDA | LOO-CV | No | Acc: 95.2% (In classifying CPT-defined groups) | CPT-defined groups yield much higher accuracy than DSM-defined groups (95.2% vs 23.4%) |
| Cheng et al., 2020 | 11-16 | 1220,  -ADHD: 799,  - Control: 421, | KSADS, CPT, Conners’ Scale | DNN (imputation), SVM (Classification) | 10-fold CV | No | Acc: 89% | High discriminative features included oppositional behaviors that rated by teachers and hyperactivity/impulsivity that rate by both parents and teachers. |
| Vaidya et al., 2020 | 8-14 | 320,  -ADHD: 84,  -Control: 139,  -ASD: 97 | ADHD rating scale, BRIEF, CBCL | SVM | No | 692 independent subjects | Acc: 77% | Three executive function subtypes can be defined, including: (a) weakness in flexibility and emotion regulation; (b) weakness in inhibition; and (c) weakness in working memory, organization, and planning. |
| Goh et al., 2021 | 18-90 | 1242,  -ADHD: 52,  - Control: 1190 | DSM-IV symptoms scale, Barkley Functional Impairment Rating Scale | RF | 10-fold CV | 20% of the original sample | R^2^: 0.46 | Important features in predicting functional impairments included difficulty organizing; does not follow through; makes careless mistakes; and difficulty  engaging in leisure activities. |

Age was reported as range. Acc: Accuracy; ADHD: Attention Deficit/Hyperactivity Disorder; ASD: Autism Spectrum Disorder; BD: Bipolar disorder; BRIEF: Behavior Rating Inventory of Executive Function; CBCL: Children Behavior Checklist; CPT: Continuous Performance Task; CV: Cross-validation; DNN: Deep neural network; DSM: The Diagnostic and Statistical Manual of Mental Disorders; KSADS: Kiddie Schedule for Affective Disorders and Schizophrenia; LOO-CV: Leave-one-out cross-validation; LDA: Linear discriminant analysis; RF: Random forest; SVM: Support vector machine.

**Supplementary Table 2**. Health record and registry studies using machine learning.

| **Study** | **Age (Year)** | **Sample Size** | **Features** | **Machine Learning Model** | **Cross-validation Method** | **Independent Testing Dataset** | **Model Performance** | **Key Findings** |
| --- | --- | --- | --- | --- | --- | --- | --- | --- |
| Fouladvand et al., 2020 | 13-18 | 11624,  -ADHD with no SUD: 5812,  -ADHD with SUD: 5812 | Medical and family history | LSTM | 20-fold CV | 10% of the original sample | Acc: 84%, Precision: 0.96, Recall: 0.72,  F1-score: 0.82 | ADHD medication initiation during adolescence was significant predictor for SUD in children with ADHD. |
| Garcia-Argibay et al., 2022 | 3-18 | 238,696,  -ADHD: 12,893,  -Control: 225,803 | Medical and Family History | LR, RF, Gradient Boosting, XGBoost, Naïve Bayes, Elastic Net, DNN | 10-fold CV | 20% of the original sample | Acc: 68.4%,  AUC: 0.753,  Sen: 71.66%,  Spe: 65% | The top 5 features contributing to classification were having a parent with criminal convictions, male sex, having a relative with ADHD, number of academic subjects failed, and speech/learning disabilities. |
| Zhang-James et al., 2020 | 18-19 | 19184,  -ADHD with SUD: 8.8% | Medical and family history | Random Forest | 15% of the original data | 15% of the original sample | AUC: 0.73 | Crime behavior, Early diagnosis of ADHD, ADHD medication, family income, and anxiety diagnosis were significant predictors for SUD in children with ADHD |

Age was reported as range. Acc: Accuracy; ADHD: Attention Deficit/Hyperactivity Disorder; AUC: Area under the receiver-operating-characteristic curve; CV: Cross-validation; DNN: Deep neural network; LR: Logistic regression; LSTM: Long short-term memory; RF: Random forest; Sen: Sensitivity; Spe: Specificity; SUD: Substance use disorder.

**Supplementary Table 3**. Neuroimaging studies using machine learning.

| **Study** | **Age (Year)** | **Sample Size** | **Features** | **Machine Learning Model** | **Cross-validation Method** | **Independent Testing Dataset** | **Model Performance** | **Key Findings** |
| --- | --- | --- | --- | --- | --- | --- | --- | --- |
| **Structural MRI and DTI studies** | | | | | | | | |
| Chang et al. 2012 | Children: 12.12±2.95 | 436,  -ADHD: 210,  -Control: 226 | Local binary pattern | SVM | 10-fold CV | No | Acc: 69.95% | The local binary pattern in gray matter was more discriminative than white matter and CSF for ADHD |
| Igual et al. 2012 | Children:10.8±2.9 | 78,  -ADHD: 39,  -Control: 39 | Dissociated Dipoles in segmented caudate. | SVM | 5-fold CV | No | Acc: 94.04%,  Sen: 96.21%,  Spe: 91.23% | Anatomical features in caudate was able to differentiated children with ADHD and controls. |
| Lim et al. 2013 | 10-18 | 77,  -ADHD: 29,  -Control: 29,  -ASD: 19 | Structural image voxels | GPC | LOO-CV | No | Acc: 79.3% (ADHD vs control), 85.2% (ADHD vs ASD), 77.1% (ADHD vs ASD & Controls),  Sen: 75.9%, 86.2%, 79.3%,  Spe: 82.8%, 84.2, 75.0% | Ventrolateral/premotor fronto-temporo-limbic regions were highly discriminative for ADHD. |
| Peng et al. 2013 | 9-14 | 152,  -ADHD: 59,  -Control: 93 | ROI-based anatomical features (thickness, area, volume, folding index, curvature) | Extreme Learning Machine | LOO-CV | No | Acc: 90.18% | Features in frontal, temporal, occipital, insular regions were most discriminative for ADHD. |
| Johnston et al. 2014 | Children:12.5±2.3 | 68,  -ADHD: 34,  -Control: 34 | Structural image voxels | SVM | Nested CV: LOO-CV | No | Acc: 93% (White matter), 63% (gm voxel), 81% (combined),  Sen: 100%, 68%, 74 %,  Spe: 85%,59%,88% | White matter in brainstem was most discriminative features for ADHD.  White matter image is more discriminative than gray matter image. |
| Wang et al. 2018 | Children:11.03±2.73 | 71,  -ADHD: 36,  -Control: 35 | Interregional morphological connectivity | SVM | LOO-CV | No | Acc: 74.65%,  Sen: 75.0%,  Spe: 74.29% | Morphological connectivity in insula, caudal anterior cingulate cortex, frontal pole, postcentral cortex were most discriminative between ADHD and controls |
| Oztekin et al. 2021 | 4-7 | 162,  -ADHD: 87,  -Control: 75 | Behavioral/cognitive measures of executive function, ROI-based cortical thickness and subcortical volume | SVM | 5-fold CV | No | Acc: 94.4% | The cortical thickness in left hemisphere were more discriminative than right hemisphere for ADHD (Acc:61% vs 50%)  The teacher’s rating of executive functions were more discriminative than cortical thickness for ADHD (Acc:94.4% vs 61%) |
| Zhang-James et al. 2021 | 4-63 | 4042,  -ADHD: 2192,  -Control: 1850 | ROI-based cortical thickness, surface area, and subcortical volume | Neural Network | 15% of the original sample | 0.15% of the original sample | AUC: 0.64 (in children), 0.56 (in adult), 0.6 (in combined group), | Anatomical features were more discriminative for ADHD in children than in adult. The high discriminative features showed similarity between children and adults. |
| Elliott et al. 2022 | 12-24 | 155,  -ADHD: 74,  -Control: 81 | Tractography | LASSO | LOO-CV | No | Spearman’s correlation: 0.17 | Increased tract integrity between the limbic striatal and SN/VTA regions predicted greater impulsivity, while increased integrity between executive striatal and SN/VTA regions predicted reduced impulsivity. |
| **Task-based fMRI Studies** | | | | | | | | |
| Hart et al. 2014a | 10-17 | 60,  -ADHD: 30,  -Control: 30 | Voxel-level brain activation during stop-signal task. | GPC | LOO-CV | No | Acc: 77%,  AUC: 0.81,  Sen: 90%,  Spe: 63% | Lateral prefrontal,  striatal, and temporo-parietal areas were predictive for controls, ventromedial fronto-limbic regions were predictive for ADHD. |
| Hart et al. 2014b | 10-17 | 40,  -ADHD: 20,  -Control: 20 | Voxel-level brain activation during a fine-temporal discrimination task | GPC | Nested CV: LOO-CV | No | Acc: 75%,  AUC: 0.73,  Sen: 80%,  Spe: 70% | Brain activation in inferior and dorsolateral prefrontal, insula, and parietal cortices, and the basal ganglia, anterior cingulate, and cerebellum were discriminative between ADHD and controls |
| Iannaccone et al. 2015 | 12-16 | 40,  -ADHD: 20,  -Control: 20 | Voxel-level brain activation during Go/NoGo task, structural image. | SVM | LOO-CV | No | Acc: 77.78%,  Sen: 77.78%,  Spe: 77.78% | The most predictive regions for ADHD include left superior temporal gyrus, left occipital gyrus, bilateral posterior cingulate cortex, bilateral cerebellum, bilateral inferior temporal lobe, bilateral medial superior frontal gyrus, right parahippocampus and brainstem. |
| Wolfers et al. 2016 | 7-27 | 415,  -ADHD: 184,  -Control: 128,  -Unaffected Siblings: 103 | Voxel-level brain activation during stop-signal task. | GPC | LOO-CV | No | AUC: 0.65 (ADHD vs unaffected siblings), 0.64 (ADHD vs controls), 0.59 (unaffected siblings vs controls) | Functional patterns in frontolateral, superior temporal and inferior parietal regions were associated with risk for ADHD |
| **Resting State fMRI Studies** | | | | | | | | |
| Bohland et al., 2012 | 7-21 | 776 (ADHD-200),  -ADHD: 285,  -Control: 491 | Functional connectivity, cortical thickness, subcortical volume | SVM | 2-fold CV | 168 independent subjects | Acc: 76%,  AUC: 0.78 | Including neuroimaging features increased generalizability of the classification models. |
| Brown et al. 2012 | 7-21 | 776 (ADHD-200),  -ADHD-C:141,  -ADHD-IA: 98,  -Control: 491 | Average activity, frequency, functional connectivity | Logistic Classifier, SVM | 10-fold CV | 171 independent subjects | Acc: 62.52% | Personal characteristics including age and sex were beneficial in construct neuroimaging-based classifier for ADHD. |
| Cheng et al., 2012 | 7-21 | 244 (ADHD-200),  -ADHD: 101,  -Control: 143 | Functional connectivity, fractional amplitude of low frequency fluctuations, regional  homogeneity | SVM | LOO-CV | No | Acc: 76.15%,  Sen: 63.27%,  Spe: 85.11% | Features associated with frontal region and cerebellum were most discriminative for ADHD. |
| Eloyan et al., 2012 | 7-21 | 776 (ADHD-200),  -ADHD: 285,  -Control: 491 | Functional connectivity | SVM, RF, gradient boosting | 184 subjects from the original sample | 195 independent subjects | Acc: 61%,  Sen: 94%,  Spe: 21% | Functional connectivity in motor network was most discriminative for ADHD. |
| Fair et al., 2012 | 7-14 | 647 (ADHD-200),  -ADHD-C: 112,  -ADHD-IA: 80,  -Control: 455 | Functional connectivity | SVM | LOO-CV | No | Acc: 77% (ADHD-C vs control), 80.8% (ADHD-IA vs control), 69.2% (Three groups) | Discriminative features for ADHD-C were associated with default mode network, insula, and sensorimotor network.  Discriminative features for ADHD-IA were associated with frontal region, cerebellum, and sensorimotor network |
| Dey et al., 2014 | 8-26 | 366 (ADHD-200),  -ADHD: 135,  -Control: 231 | Functional Network | SVM | LOO-CV | 121 independent subjects | Acc: 73.55% | Sex is a significant factor for accurate classification of ADHD |
| Wang et al., 2017 | Children:11.98±1.88 | 143 (ADHD-200),  -ADHD: 74,  -Control: 69 | Phase synchronization, sample entropy, and functional connectivity of intrinsic connectivity network | LASSO | LOO-CV | No | MAE: 3.3 (Inattention), 4.08 (Impulsivity) | The sample entropy contributed most to the prediction of both inattention and impulsivity |
| Zhao et al., 2022 | 7-21 | 603 (ADHD-200),  -ADHD: 260,  -Control: 343 | Functional connectivity | Graph CNN | 10-fold CV | No | Acc: 72%,  Sen: 72.2%,  Spe: 71.6% | Discriminative feature for ADHD were associated with temporal lobe, frontal lobe, and cerebellum. |
| **EEG Studies** | | | | | | | | |
| Mueller et al. 2011 | 20-50 | 150,  -ADHD: 75,  -Control: 75 | ERP | SVM | 10-fold CV | 17 independent ADHD subjects | Acc: 91%,  Sen: 91%,  Spe: 91% | Both latency and amplitude measure were important in differentiate ADHD and controls |
| Tenev et al. 2014 | 18-50 | 117,  -ADHD: 67  -Control: 50 | Power of 4 frequency bands during 4 conditions | SVM | 10-fold CV | No | Acc: 82.3% | Classification models that combine data from multiple conditions yield better performance than single condition. |
| Helgadóttir et al. 2015 | 5-14 | 661,  -ADHD: 310,  -Control: 351 | Power spectrum | SVM | 10-fold CV | No | Acc: 76%,  AUC: 0.73 | Age was an important feature in EEG-based classification of ADHD. |
| Biederman et al. 2017 | Adult: 30.06±10.76 | 63,  -ADHD: 34,  -Control: 29 | ERP | SVM | 10-fold CV | No | AUC: 0.92,  Sen 86%,  Spe: 95% | ERP during Go condition was more discriminative for ADHD than ERP during NoGo condition. |
| Vahid et al. 2019 | Children: 10.9±2.4 | 144,  -ADHD-C: 48,  -ADHD-IA: 52,  -Control: 18 | Time series of standardized current density | CNN | LOO-CV | No | Acc: 69% (all ADHD vs control), 83% (ADHD-IA vs controls), 80% (ADHD-C vs controls) | - Neurophysiological processes indicating attentional  selection associated with superior parietal cortical areas were the most important for classification between ADHD and controls.  - The model was not able to differentiate ADHD subtypes. |
| Kim et al., 2021 | 18-45 | 79,  -ADHD: 34,  -Control: 45 | Mismatch negativity | SVM | LOO-CV | No | Acc: 81.01%,  Sen: 82.35%,  Spe: 80% | Important features included mismatch negativity amplitude at FC4 and mismatch negativity source activities in the bilateral anterior cingulate cortex, inferior frontal gyrus, rectal gyrus, subcallosal gyrus, extra nuclear, orbital gyrus, and uncus, and right superior temporal gyrus. |
| Chang et al. 2022 | Children: 6.4±1.9 | 60,  -ADHD: 30,  -Control: 30 | Time-series of different channels and different frequency band | LSTM | Nested CV: 10-fold CV, LOO-CV | No | Acc: 90.5%,  Sen: 90%,  Spe: 91% | Beta power activity of the  O1 and O2 sites contributed the most to the classifications, subjects in the ADHD group exhibited decreased beta power compared to controls |
| **fNIRS Studies** | | | | | | | | |
| Gu et al., 2018 | 6-9 | 50,  -ADHD: 25,  -Control: 25 | Mean amplitude of oxygenated hemoglobin concentration during 1-back task. | SVM | LOO-CV | No | Acc: 86%,  Sen: 84%,  Spe: 88% | The important features included activations in bilateral dorsolateral prefrontal cortex, inferior medial prefrontal cortex, right posterior  prefrontal cortex, and right temporal cortex. |
| Yasumura et al. 2020 | children:10.28±2.2 | 216,  -ADHD: 108,  -Control: 108 | Prefrontal cortex activation during reverse Stroop task and performance of reverse Stroop task. | SVM | 3-fold CV | 99 independent subjects (ADHD: 62, Controls: 37) | Acc: 86.25%,  AUC: 0.898,  Sen: 88.71%  Spe: 83.78% | Including fNIRS data during reverse Stroop task yield better classification accuracy than using only the performance of the task. |
| **Multimodal Neuroimaging Studies** | | | | | | | | |
| Luo et al. 2020 | Adult: 24.4 ± 2.1 | 72,  -ADHD-persister: 18,  -ADHD-remitter: 18,  -Control: 36 | sMRI, DTI, fMRI | Ensemble Learning | Nested CV: LOO-CV, 5-fold CV | No | AUC: 0.89 (ADHD vs controls),  0.9 (ADHD persisters vs remitters) | Important brain regions for classification included: right inferior frontal gyrus, right middle frontal gyrus, inferior parietal gyrus, and right amygdala. |
| Owens et al., 2021 | 9-10 | 11875 (ABCD Dataset),  -ADHD: 727,  -Control: 18 | fMRI, sMRI | Elastic Net | Nested CV: 20-fold CV, 5-fold CV | 20% of the original sample | R2:0.8% (sMRI), 1.9% (n-back),0.1% (SST) | Morphometry had higher predictive value for ADHD symptoms than activation during tasks. |
| Zhou et al. 2021 | 9-10 | 232 (ABCD Dataset),  -ADHD: 116,  -Control: 116 | rsMRI, sMRI, DTI | SVM | Nested CV:10-fold CV, 5-fold CV | No | Acc: 64.3%,  AUC: 0.698,  Sen: 60.9%,  Spe: 67.6% | Important features included functional connectivity in default mode network, attention network, auditory network, sensorimotor mouth network, thalamus, and cerebellum during rsMRI, and morphometry in basal ganglia. |

Age was reported as range or mean ± standard deviation. ABCD: Adolescent Brain Cognitive Development; Acc: Accuracy; ADHD: Attention Deficit/Hyperactivity Disorder; ADHD-C: ADHD Combine; ADHD-IA: ADHD Inattentive; ASD: Autism Spectrum Disorder; AUC: Area under the receiver operating characteristic curve; CNN: Convolutional neural network; CPT: continuous performance task; CV: Cross-validation; DNN: deep neural network; DTI: diffusion tensor imaging; EEG: electroencephalogram; ERP: event-related potential; fMRI: functional magnetic resonance imaging; fNIRS: functional near-infrared spectroscopy; GPC: gaussian process classifier; LASSO: least absolute shrinkage and selection operator; LOO-CV: Leave-one-out Cross-validation; LR: Logistic regression; LSTM: long short-term memory; MAE: Mean Absolute Error; RF: Random forest; ROI: Region of interest; rsMRI: resting-state magnetic resonance imaging; Sen: sensitivity; sMRI: structural Magnetic Resonance Imaging; Spe: specificity; SVM: support vector machine.

**Supplementary Table 4**. Genetic studies using machine learning.

| **Study** | **Age (Year)** | **Sample Size** | **Features** | **Machine Learning Model** | **Cross-validation Method** | **Independent Testing Dataset** | **Model Performance** | **Key Findings** |
| --- | --- | --- | --- | --- | --- | --- | --- | --- |
| van der Meer et al., 2017 | Children and Adults:  17.1±3.4 | 686,  -ADHD: 281,  -Control: 405 | SNPs | RF Regression | Random sample CV | No | R^2^: 12.5% | -Important predictors included SNPs in or near TERT, NPSR1, ESR1, GABRA6, PER3, NR3C2 and DRD4. |
| Yoo et al., 2020 | 6-17 | 94,  -ADHD: 47,  -Control: 47 | Polygenic risk score, rsMRI, DTI, sMRI | RF,  RF regression | LOO-CV | 36 independent subjects | Acc: 85.1%,  AUC: 0.877,  Sen: 58.1%,  Spe: 58.1%,  R^2^: 0.18 (for ADHD symptoms) | -Morphological changes across insula, sensory/motor, and inferior frontal cortex were found as key predictors.  -The effect of polygenic risk score on classification is minimal. |
| Liu et al., 2021a | Children and Adults: 22.2±3.3 | 524,  -ADHD: 116,  -Control: 408 | CNV count | DNN | 2-fold CV | 351 Independent Subjects | Acc: 78% | -High predictive genomic regions included genes GRM1 and GRM8. |
| Liu et al., 2021b | Patients (Children): 10.9±4.6,  Control (Adults): 40.7±12.5 | 1983,  -ADHD: 1033,  -Control: 950 | SNPs | DNN | 5% of the original sample | 20% of the original sample | Acc: 90.18%, AUC: 0.957,  Sen: 89.8%,  Spe: 90.55% | -Highly discriminative genes included NRG3, TENM4, LIG4, MDGA2, BMP2,  EPHA5, PHA7, LPAR1, and TLR4. |
| Sudre et al. 2021 | Children: 8.7±3.0 (baseline), 13.3±3.1 (follow-up) | 362,  -Improver: 110,  -Stable: 63,  -Worsening: 60,  -Control: 129 | Demography, DTI, Polygenetic risk score, Neurocognitive performance | RF | 3-fold CV | 10% of the original sample | AUC: 0.77-0.94 (Between group pairs),  Sen: 58-91%,  Spe: 58-100% | -Cognitive performance features were most predictive for ADHD and controls, while the polygenic risks were most predictive for the worsening of the ADHD symptoms. |
| Cervantes-Henriquez et al., 2022 | 6-60 | 408  -ADHD: 236,  -Control: 172 | SNPs | LR, RF, SVM, XGBoost | 10-fold CV | 30% of the original sample | Acc: 82% | Gene regions SNAP25, ADGRL3, and DRD4 were significantly contributed to the prediction of inattentive, hyperactive, or impulsive symptoms |
| Wang et al., 2022 | 6-16 | 228,  -ADHD: 145,  -Control: 83 | MicroRNA level change | SVM | 10-fold CV | No | AUC: 0.966,  Sen: 96%,  Spe: 94.2% | miR-140-3p, miR-27a-3p, miR-486-5p, and miR-151-5p showed differential trends between ADHD and controls. |

Age was reported as range or mean ± standard deviation. Acc: accuracy; ADHD: attention deficit/hyperactivity disorder; CNV: Copy number variants; CV: cross-validation; DNN: deep neural network; DTI: diffusion tensor imaging; LOO-CV: leave-one-out cross-validation; LR: logistic regression; LSTM: long short-term memory; RF: Random forest; rsMRI: resting-state magnetic resonance imaging; Sen: sensitivity; sMRI: structural Magnetic Resonance Imaging; SNP: single nucleotide polymorphism; Spe: specificity; SVM: support vector machine.

**Supplementary Table 5**. Treatment studies using machine learning.

| **Study** | **Age** | **Sample Size** | **Features** | **Machine Learning Model** | **Cross-validation Method** | **Independent Testing Dataset** | **Model Performance** | **Key Findings** |
| --- | --- | --- | --- | --- | --- | --- | --- | --- |
| Johnston et al., 2015 | Children:11.19±2.39 | 43,  -Good response: 30,  -Poor response: 13 | Demography, clinical variables, neuropsychological performance | SVM | Nested CV: LOOCV | No | Acc: 76.7%,  Sen: 54%,  Spe: 87% | Important features for predicting the efficacy of MPH included performance of Go/NoGo task and comorbid conduct disorder. |
| Kim et al., 2015 | Children:9.5±2.6 | 78,  -Good response: 48,  -Poor response: 30 | Demography, clinical information, neuropsychological performance, genetic/environmental/neuroimaging measures | SVM, DT, RF, Ridge Regression | 10-fold CV | No | Acc: 84.6%,  AUC: 0.84 | Important predictors included age, weight, ADRA2A MspI and DraI polymorphisms, lead level, Stroop color word test performance, and oppositional symptoms of Disruptive Behavior Disorder rating scale |
| Fouladvand et al., 2020 | 13-18 | 11624,  -ADHD with no SUD: 5812,  -ADHD with SUD: 5812 | Medical and family history | LSTM | 20-fold CV | 10% of the original sample | Acc: 84%, Precision: 0.96, Recall: 0.72,  F1-score: 0.82 | ADHD medication initiation during adolescence was significant predictor for SUD in children with ADHD. |
| Yoo et al., 2020 | children:9.5±2.6 | 83,  -With side effect: 10,  -No side effect: 73 | Demography, neuropsychological performance, clinical/behavioral measures, genetic variables, structural connectivity | SVM, DT, Ridge Regression | 10-fold CV | 36 independent subjects | Acc: 86.1%,  AUC: 0.92,  Sen: 87%,  Spe: 86% | Important features for predicting sleep problems after MPH included inattention symptoms, CPT response time, DAT1, ADRA2A DraI, and SLC6A2 A3081T Polymorphisms, and structural connectivity between frontal and striatal regions. |
| Zhang-James et al., 2020 | 18-19 | 19184,  -ADHD with SUD: 8.8% | Medical and family history | RF | 15% of the original data | 15% of the original sample | AUC: 0.73 | Crime behavior, Early diagnosis of ADHD, ADHD medication, family income, and anxiety diagnosis were significant predictors for SUD in children with ADHD |
| Chang et al., 2021 | 6-42 | 79,  -Good response: 63,  -Poor response: 16 | Gray matter volumetric image | SVM | Nested CV:5-fold CV, LOO-CV | No | Acc: 87.4%,  AUC: 0.88,  Sen: 81.3%,  Spe: 93.7% | Important features for predicting the efficacy of MPH included volumetric information in left putamen and frontoparietal regions, bilateral precuneus and occipital regions, and bilateral posterior/inferior cerebellum. |
| Faraone et al., 2021 | Children:11±3.2 | 774,  -Good response: 344,  -Poor response: 430 | Demography, ADHD rating scale-5 | LASSO, SVM, RF, Gradient Boosting | 50-fold CV | 20% of the original sample | AUC: 0.76,  Sen: 75%,  Spe: 74% | Early responses (after 2 weeks) to SPN-812 (viloxazine extended-release), measured by ADHD rating scale-5, was able to predict efficacy outcome at week 6. |

Age was reported as range or mean ± standard deviation. Acc: accuracy; ADHD: attention deficit/hyperactivity disorder; ASD: autism spectrum disorder; BD: bipolar disorder; CAARS: Conners' Adult ADHD Rating Scale; CBCL: Children Behavior Checklist; CPT: Continuous Performance Task; CV: cross-validation; DNN: deep neural network; DT: decision tree; LOO-CV: leave-one-out cross-validation; LASSO: least absolute shrinkage and selection operator; LSTM: long short-term memory; MPH: methylphenidate; RF: Random forest; Sen: sensitivity; Spe: specificity; SVM: support vector machine; .
